# Impact of second-layer coverages on complication rates in primary tubularized incised plate urethroplasty (TIPU) for distal and midpenile hypospadias repair: a systematic review

**DOI:** 10.1007/s00383-025-06134-3

**Published:** 2025-08-01

**Authors:** Marta Pezzoli, Mattia Lo Re, Virginia Carletti, Lorenzo Masieri, Alberto Mantovani

**Affiliations:** 1 Department of Urology, University of Florence, Careggi Hospital, Florence, Italy; 2 Department of Pediatric Urology, University of Florence, Meyer Children Hospital, Florence, Italy

**Keywords:** Hypospadias repair, Urethrocutaneous fistula, Second layer, Dorsal dartos, Tunica vaginalis

## Abstract

This systematic review assesses the impact of different second-layer coverage techniques on complication rates following primary tubularized incised plate urethroplasty (TIPU) for distal and midpenile hypospadias. A systematic search of PubMed, EMBASE, Cochrane Central, and Scopus was conducted in August 2024. Studies were included if they reported outcomes of single- or double-layer neourethral coverage in primary TIPU for distal or midpenile hypospadias. A narrative synthesis was performed due to study heterogeneity. Forty studies met inclusion criteria. In distal hypospadias, single-layer coverage yielded urethrocutaneous fistula (UCF) rates below 10% in most cases. Meatal stenosis reached 33.3% but was uncommon with dorsal dartos (DD) flaps. Double-layer coverage, especially with double DD flaps, showed lower UCF rates (0–12)% and minimal stenosis. For midpenile hypospadias, single-layer coverage showed higher UCF rates (0–36.4%), with DD flaps performing worse (12.5–36.4%) than tunica vaginalis (TV) flaps (0–3.1%). Double-layer techniques consistently reduced UCF to < 5%, with double DD flaps showing no fistula or stenosis. In conclusion, second-layer coverage, particularly double layer, reduces complications in TIPU. The DD flap remains most commonly used due to its accessibility, while the technically demanding TV flap shows promising results. Further high-quality data are needed to identify the optimal technique.

## Introduction

Hypospadias is a congenital penile anomaly defined by an ectopic ventral urethral meatus, which can be distinguished as distal, midpenile, or proximal based on its position. Distal and midpenile hypospadias are the predominant presentations [1]. Surgical correction seeks to reconstruct a penis that is both functionally and aesthetically acceptable [1], with tubularized incised plate urethroplasty (TIPU) being the most commonly adopted technique for the primary repair of distal and midpenile hypospadias [2].

The apposition of a second layer over the neourethra is an essential factor of surgical success, strongly recommended to avert complications, such as urethrocutaneous fistula (UCF) or wound dehiscence. Various tissues, such as dartos, tunica vaginalis (TV), Buck’s fascia, and spongiosa, have been employed as single or double-layer reinforcement over the neourethra to mitigate postoperative complications [3]. Despite the extensive implementation of various coverage strategies, debate continues concerning the most effective method, especially the efficacy of single-layer compared to double-layer reinforcement [4].

This systematic review seeks to assess the impact of different second layers on complication rates after primary TIPU for distal and midpenile hypospadias. By integrating data from studies that distinguish between these coverage strategies, we want to elucidate which technique yields improved outcomes and determine the most effective tissue types for minimizing complications.

## Methods

### Inclusion and exclusion criteria

#### Study design

This systematic review encompassed various study designs, including randomized controlled trials (RCTs) and non-randomized prospective and retrospective studies. The exclusion criteria ruled out case reports, case series involving fewer than five patients, letters to the editor, editorials, comments, conference abstracts, animal studies, and review articles. In addition, studies that were not in English or for which full-text articles were unavailable were excluded.

#### Patient population and intervention

The review targeted studies on male patients with distal and midpenile primary hypospadias undergoing TIPU, utilizing either a single or double layer for neo-urethral coverage. All forms of urethral coverage were eligible for inclusion. Studies focusing on proximal and penoscrotal hypospadias, or those that did not differentiate between distal, midpenile, and proximal hypospadias in their outcomes, were excluded. The review also excluded studies involving re-do urethroplasties, non-TIPU repairs, fistula repairs, two-stage repairs, or the absence of urethral coverage. Studies that did not report the incidence of postoperative UCF were excluded from the analysis.

#### Outcome

The primary outcome assessed was the rate of postoperative complications, which included UCF, meatal/urethral stenosis, wound and glans dehiscence, and preputial skin necrosis.

#### Data extraction

Data extracted from each included study comprised the following: the first author’s name, publication year, study design, number of patients, patient age, location of the hypospadic defect, use of a single or double layer (with descriptions of each layer), post-op catheter size, duration of catheterization, follow-up period, and the incidence of each complication.

#### Literature search

**Fig. 1:**
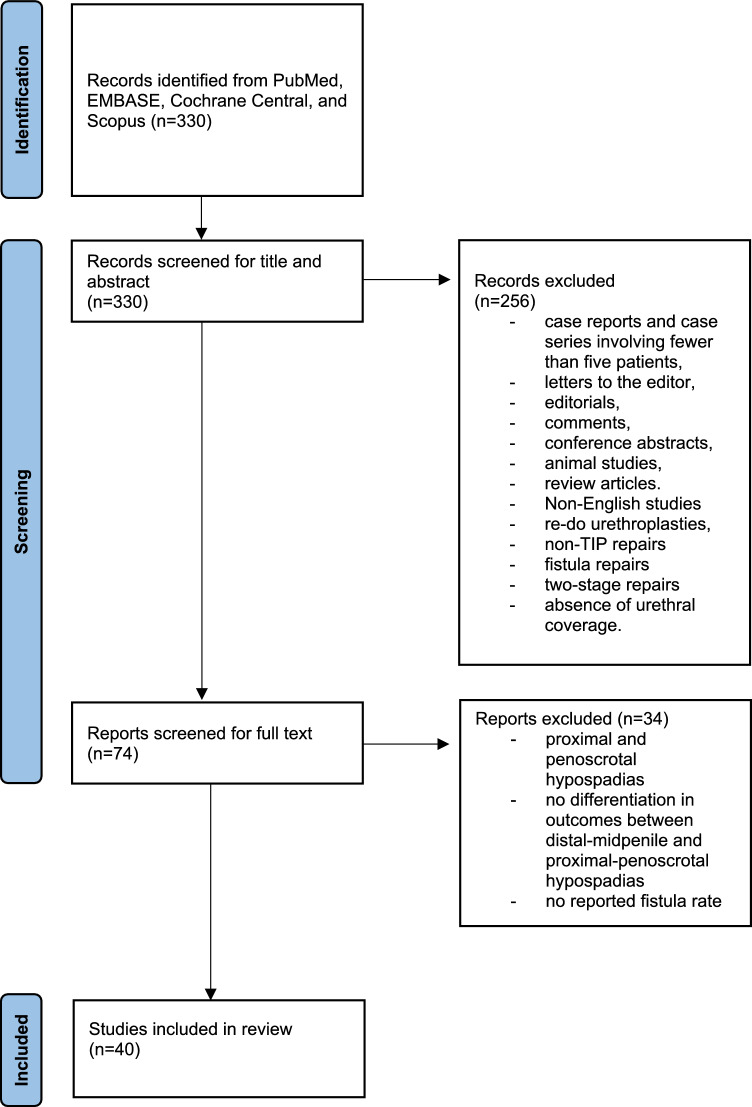
PRISMA flowchart

In August 2024 a thorough literature search was performed across four databases: PubMed, EMBASE, Cochrane Central, and Scopus. The search strategy used in PubMed was: (Hypospadias) AND ((layer) OR (single layer) OR (double layer)) AND ((flap) OR (tunica vaginalis) OR (dartos)). This search returned 330 articles. After screening titles and abstracts, 74 articles were shortlisted for further evaluation. Upon full-text review, 40 articles met the inclusion criteria and were incorporated into the systematic review. The selection process is outlined in a PRISMA flow diagram, as shown in Fig. 1.

The protocol for this systematic review was registered in the International Prospective Register of Systematic Reviews (PROSPERO), registration number: CRD420251028867.

#### Data analysis

A data collection form was developed a priori and was used to retrieve and collect data from each included study. As only few RCTs were identified, a meta-analysis and quantitative analysis of data were considered not appropriate; therefore, a narrative synthesis of findings was conducted instead.

#### Ethical statement

This systematic review was conducted using published and publicly available information. As no human subjects were involved, this study was classified as non-human subject research, and Institutional Review Board (IRB) approval was not required.

## Evidence synthesis

### Characteristics of included studies

A total of 40 studies were identified, which addressed the postoperative complications of primary repair of distal and midpenile hypospadias after TIPU repair, with a single or double layer for neo-urethral coverage. These studies included 5920 patients. Half of these studies were published within the past decade, and all of them were published between 2002 and 2024. The study design was retrospective for 18 articles, prospective for 14, and RCTs for 8.

Patients’ median follow-up durations varied from 4.5 to 57.6 months, while their mean ages ranged from 0.9 to 12.2 years.

Fourteen articles addressed exclusively distal hypospadias, ten articles addressed both distal and midpenile hypospadias with a distinction in outcomes, and sixteen articles addressed both distal and midpenile hypospadias without a distinction in outcomes.

In eleven articles, all patients received the same type of single or double layer. In the remaining articles, patients received either the same type of layer in single or double format, single layers of a different type, double layers of a different type, or a combination of single and double layers using different tissues.

For this reason, we grouped together all patients in the same study who received the same type of layer for neo-urethral coverage and conducted the analysis by comparing these homogeneous groups.

Details of included articles are available in Table 1.

**Table 1:** Included articles

First author, year	Homogeneous groups	N° patients	Localization of the meatus	Single/double layer	Type of Layer	Fistula rate (%)	Stenosis rate (%)
Hamid, 2024 [13]	1	84	Distal	S	BF	2,4	3,6
	2	80	Distal	S	DD	7,5	10,0
Al-Taher, 2023 [29]	1	116	Distal–midpenile	S	DD	22,4	8,6
	2	47	Distal–midpenile	D	DD	0,0	17,0
Asad, 2023 [39]	1	296	Distal–midpenile	S	DD	7,1	5,1
Li, 2022 [28]	1	142	Distal	D	DEDP	2,1	2,1
	2	95	Midpenile	D	DEDP	4,2	3,2
Yiğit, 2022 [38]	1	45	Distal–midpenile	S	VD	22,2	11,1
	2	44	Distal–midpenile	S	DD	11,4	4,5
Okumuş, 2022 [16]	1	242	Distal	D	Spongioplasty + DD	3,3	0,0
	2	231	Distal	S	DD	6,5	0,4
Maheshwari, 2022 [23]	1	12	Distal	S	Spongioplasty	0,0	8,3
	2	5	Midpenile	S	Spongioplasty	0,0	0,0
Dahal, 2022 [36]	1	37	Distal–midpenile	S	VD	24,3	8,1
	2	37	Distal–midpenile	D	VD + BF	5,4	2,7
Naumeri, 2021 [18]	1	21	Distal	S	DD	23,8	0,0
	2	27	Distal	D	DD	3,7	0,0
	3	9	Midpenile	S	DD	22,2	0,0
	4	3	Midpenile	D	DD	0,0	0,0
Verma, 2021 [5]	1	15	Distal	S	DD	20,0	6,7
	2	15	Distal	S	Spongioplasty	26,7	33,3
Sengol, 2021 [20]	1	42	Distal	S	DD	7,1	0,0
	2	32	Midpenile	S	TV	3,1	0,0
	3	8	Distal	S	Spongioplasty	0,0	12,5
	4	5	Midpenile	S	Spongioplasty	0,0	0,0
Han, 2020 [37]	1	11	Distal–midpenile	S	VD	0,0	0,0
	2	11	Distal–midpenile	D	VD + TV	0,0	0,0
Maily, 2020 [17]	1	41	Distal	S	DD	4,9	2,4
	2	44	Distal	S	VD	6,8	2,3
Mahmoud, 2019 [33]	1	90	Distal–midpenile	S	PRP	10,0	1,1
	2	90	Distal–midpenile	S	VD	13,3	1,1
Omar, 2018 [34]	1	37	Distal–midpenile	S	PPF	5,4	2,7
Basavaraju, 2017 [19]	1	8	Distal	S	TV	0,0	0,0
	2	6	Midpenile	S	TV	0,0	0,0
	3	21	Distal	S	DD	9,5	4,8
	4	11	Midpenile	S	DD	36,4	0,0
	5	21	Distal	S	VD	14,3	0,0
Jia, 2016 [32]	1	185	Distal–midpenile	S	DD	2,7	1,1
	2	171	Distal–midpenile	S	VD	2,9	1,8
Bertozzi, 2016 [43]	1	127	Distal–midpenile	D	DD	2,4	2,4
Demir, 2015 [24]	1	27	Distal	D	DD	0,0	11,1
Bhat, 2014 [22]	1	81	Distal	S	Spongioplasty	2,5	0,0
	2	12	Midpenile	S	Spongioplasty	16,7	0,0
Thomas, 2014 [40]	1	107	Distal–midpenile	S	DD	6,5	0,0
Babu, 2013 [26]	1	36	Distal	D	PT/spongioplasty + DD	8,3	8,3
	2	26	Midpenile	D	PT/spongioplasty + DD	30,8	3,8
	3	21	Midpenile	D	PT/spongioplasty + TV	4,8	4,8
El-Shazly, 2013 [15]	1	21	Distal	S	VD	9,5	
	2	16	Midpenile	S	VD	6,3	
	3	13	Distal	S	GST	38,5	
	4	13	Midpenile	S	GST	23,1	
Maarouf, 2012 [7]	1	48	Distal	D	DD	0,0	2,1
	2	52	Distal	S	DD	7,7	1,9
Safwat, 2012 [8]	1	28	Distal	S	DD/VD	7,1	3,6
	2	30	Distal	D	DD	0,0	0,0
Bertozzi, 2011 [42]	1	394	Distal–midpenile	D	DD	1,0	2,8
Bilici, 2011 [31]	1	75	Distal–midpenile	S	DD	8,0	2,7
	2	86	Distal–midpenile	D	Spongioplasty + DD	0,0	3,5
Essam, 2011 [44]	1	40	Distal–midpenile	D	DD	2,5	10,0
Yildiz, 2010 [6]	1	26	Distal	S	DD	23,1	3,8
	2	53	Distal	D	DD	0,0	3,8
Tabassi, 2010 [30]	1	29	Distal–midpenile	S	TV	10,3	10,3
Abolyosr, 2010 [27]	1	98	Distal	D	DD	0,0	0,0
	2	56	Midpenile	D	DD	0,0	0,0
Snodgrass, 2010 [14]	1	245	Distal	S	DD	2,0	
	2	130	Distal	S	VD	2,3	
Erol, 2009 [9]	1	37	Distal	S	DD	8,1	8,1
	2	40	Distal	D	DD	0,0	0,0
Appignani, 2009 [41]	1	40	Distal–midpenile	S	DD	10,0	0,0
	2	57	Distal–midpenile	D	DD	0,0	5,3
Suoub, 2008 [11]	1	25	Distal	D	Foreskin reconstruction	12,0	0,0
	2	49	Distal	S	DD	8,2	0,0
El-Kassaby, 2008 [25]	1	764	Distal	D	DEDP	2,1	1,0
Kamal, 2005 [10]	1	54	Distal	S	DD	3,7	0,0
	2	42	Distal	D	DD	0,0	0,0
Soygur, 2005 [35]	1	60	Distal–midpenile	S	VD	8,3	10,0
Cheng, 2002 [12]	1	414	Distal	S	DD	0,0	0,2
Zhou, 2002 [21]	1	3	Distal	S	DD	0,0	
	2	8	Midpenile	S	DD	12,5	

*S* single, *D* double, *DD* dorsal dartos, *VD *ventral dartos, *TV* tunica vaginalis, *BF* Buck’ fascia, *DEDP* de-epithelized dorsal prepuce, *PPF* pedicled preputial flap, *PRP* platelet rich plasma, *PT* periurethral tissue, *GST* glanular subcutaneous tissue

### Complication rates in distal hypospadias repair

#### Single layer

Nineteen studies examined single-layer neo-urethral coverage, with seven of them implementing various tissues across different patient groups [5–23]. Consequently, 27 homogeneous groups were identified. Postoperative UCF rates varied widely, from 0% to 38.5%. Five groups recorded no fistulas, six groups showed rates below 5%, ten groups ranged from 5% to 10%, and six groups exceeded a 10% rate. Data on meatal stenosis were missing for five groups; among those with data, stenosis rates ranged from 0% to 33.3%, with 0% in seven groups, 0–5% in nine, 5–10% in four, and over 10% in two. Wound dehiscence was uncommon, observed in six groups with rates from 1.2% to 6.3%. Skin necrosis was rare, appearing in only two groups at rates of 2.4% and 3.6%.

##### Dorsal dartos (DD) flap

The primary single-layer applied was the dorsal dartos (DD) flap, used across 15 groups [5–7, 9–14, 16–21]. Among these, two groups observed no postoperative UCFs, three reported rates below 5%, and seven noted rates between 5% and 10%. Higher fistula rates were documented in the remaining groups, particularly in studies by Verma et al. [5] (20.0%), Yildiz et al. [6] (23.1%), and Naumeri et al. [18] (23.8%). Conversely, the incidence of meatal stenosis was minimal, with the highest noted in the article by Hamid et al. [13] (10.0%).

##### Alternative layers

Alternative layers were less frequently adopted. The ventral dartos (VD) flap was used in four groups [14, 15, 17, 19], showing a fistula rate from 2.3% to 14.3% and stenosis rates described in two groups at 0% and 2.3%. TV was applied in one group [19] without any postoperative fistulas or stenosis, while Buck’s fascia, also used in a single group [13], had fistula and stenosis rates of 2.4% and 3.6%, respectively. The glanular subcutaneous tissue was described in one group [15] with a fistula rate of 38.5%. Finally, spongioplasty was evaluated in four groups [5, 20, 22, 23]; three reported low fistula rates (0–2.5%) and stenosis rates ranging from 0% to 12.5%, while one group noted a fistula rate of 26.7% and a stenosis rate of 33.3%.

#### Double layer

In 13 studies, the double-layer technique was utilized, and the same tissues were used across patients in the individual studies [6–11, 16, 18, 24–28]. UCF rates ranged from 0% to 12%. Of the patient groups, seven had no fistulas, four had rates between 0% and 5%, one had a rate of 5–10%, and one exceeded 10%. Meatal stenosis rates ranged from 0% to 11.1%, with seven groups showing no cases, four groups between 0% and 5%, one group between 5% and 10%, and one group with more than 10%. Wound dehiscence, observed in four groups, had rates from 2.1% to 3.5%, while skin necrosis did not occur in any group.

##### Double dorsal dartos (DD) flap

The double DD flap was the most used, applied in 8 groups [6–10, 18, 24, 27]. In this cohort, seven groups reported no postoperative fistulas, while only one group observed a fistula rate of 3.7%. In contrast, meatal stenosis rates were slightly elevated in three groups, ranging from 2.1% to 11.1%, while the remaining groups reported no stenosis.

##### Alternative layers

Other double-layer techniques were used less frequently. The combination of DD flap with spongioplasty or periurethral tissue was used in two groups [16, 26], showing variable rates of complications: fistula rates ranged from 3.3% to 8.3%, and stenosis rates from 0% to 8.3%. The de-epithelialized dorsal prepuce, also used in two groups [25, 28], presented low complication rates, with fistula rates of 2.1% and stenosis rates from 1% to 2.1%. Finally, foreskin reconstruction was used in one group [11], resulting in a fistula rate of 12% and no cases of stenosis.

### Complication rates in midpenile hypospadias repair

#### Single layer

Among seven studies investigating single-layer techniques for midpenile hypospadias, three applied distinct single layers across various patient groups [15, 18–23]. Consequently, ten homogeneous groups were identified. Across the groups, UCF rates ranged from 0% to 36.4%. Three groups had no fistulas, one group had rates between 0% and 5%, one group showed rates of 5–10%, and five groups exceeded 10%, with rates between 12.5% and 36.4%. In terms of meatal stenosis, three groups did not report on it, while none of the remaining groups experienced any cases. Wound dehiscence was uncommon but reported in one group, with an incidence of 9.1%. None of the groups showed cases of skin necrosis.

Several tissue types were applied for neo-urethral coverage. The DD flap was used in three studies [18, 19, 21], showing high UCF rates between 12.5% and 36.4%, with no cases of meatal stenosis reported. The VD flap, used in one study [15], showed a fistula rate of 6.3%. TV, utilized in two studies [19, 20], resulted in low fistula rates (0–3.1%) and no occurrences of stenosis. Glanular subcutaneous tissue was described in one study [15], where 23.1% of patients developed postoperative fistulas. Finally, spongioplasty alone was applied in three studies [20, 22, 23], with two reporting no fistulas or stenosis and one reporting a 16.7% fistula rate with no stenosis.

#### Double layer

Four studies examined double-layer techniques, with one study applying different layers in two patient groups [18, 26–28]. Therefore, five groups were reviewed, and UCF rates ranged from 0% to 30.8%. Two groups showed no fistulas; two groups had rates between 0% and 5%, while one group reported a rate of 30.8%. Meatal stenosis data were unavailable for two groups; the other groups reported no stenosis or rates below 5%. Wound dehiscence rates included two groups with no cases, two groups with rates below 5%, and one group at 6.3%. Skin necrosis was absent in four groups but observed in one, with an occurrence rate of 23.1%.

Various techniques were reported. The double DD flap, applied in two groups [18, 27], resulted in no cases of postoperative UCF or meatal stenosis. In the single group, where the DD flap combined with spongioplasty or periurethral tissue was used [26], the reported complication rates were 30.8% for fistulas, 23.1% for skin necrosis, and 3.8% for both stenosis and wound dehiscence. The combination of TV with spongioplasty or periurethral tissue, utilized in one group [26], resulted in lower rates of 4.8% for fistulas, stenosis, and wound dehiscence. Finally, de-epithelialized dorsal prepuce, used in one group [28], showed rates of 4.2% for fistulas, 3.2% for stenosis, and 6.3% for wound dehiscence.

### Complication rates in studies with combined outcomes for distal and midpenile hypospadias

#### Single layer

Thirteen studies reported on single-layer neo-urethral coverage, with three using distinct layers across different patient groups [29–41]. As a result, we identified 16 groups, in which UCF rates varied from 0% to 24.3%. A single group had no fistulas, two groups had rates between 0% and 5%, seven groups ranged from 5% to 10%, and six groups exceeded a 10% rate. Meatal stenosis rates spanned 0–11.1%, with 0% in three groups, 0–5% in seven, 5–10% in four, and above 10% in two. Wound dehiscence occurred in six groups at rates from 0% to 5%, while two groups experienced higher rates of 5.6% and 10.8%. Skin necrosis was noted only once, with a 2.7% rate.

##### Dorsal and ventral dartos flap

The DD flap and VD flap were used in seven [29, 31, 32, 38–41] and six groups [32, 33, 35–38], respectively. The DD flap showed variable fistula rates, ranging from 2.7% to 10% in five groups, and higher rates of 11.4% and 22.4% in studies by Yiğit et al. and Al-Taher et al. Stenosis rates with this technique were lower, ranging from 0% to 8.6%. The VD flap showed fistula rates from 0% to 24.3%, with only three groups reporting rates below 10%; meatal stenosis rates ranged from 0% to 11.1%.

##### Alternative layers

Other layers were less commonly used. TV was applied in one group [30], with both fistula and stenosis rates at 10.3%. The pedicled preputial flap, also used in a single group [34], showed a fistula rate of 5.4% and a stenosis rate of 2.7%. Finally, Mahmoud et al. applied autologous platelet-rich plasma (PRP) in one group [33], resulting in a 10% fistula rate, 1.1% stenosis rate, and 1.1% wound dehiscence rate.

#### Double layer

Eight studies documented double-layer neo-urethral coverage [29, 31, 36, 37, 41–44], showing UCF rates that ranged from 0% to 5.4%. Four groups had no fistulas, three had rates between 0% and 5%, and one reported a rate of 5.4%. Meatal stenosis rates ranged from 0% to 17%, with one group showing no cases, four groups between 0% and 5%, two between 5% and 10%, and one with a rate of 17%. Wound dehiscence was relatively rare, with three groups reporting rates from 0.5% to 2.7%. No skin necrosis was noted across any of the groups.

##### Double dorsal dartos (DD) flap

The double DD flap was the most frequently applied technique, used in five groups [29, 41–44]. Among these, two groups reported no postoperative fistulas, while the remaining groups observed fistula rates under 2.5%. In contrast, meatal stenosis rates were higher, ranging from 2.4% to 10% in four groups and reaching 17% in one group.

##### Alternative layers

The combination of DD flap with spongioplasty was applied in one group [31], resulting in no fistulas and a stenosis rate of 3.5%. The VD flap combined with Buck’ fascia, also used in one group [36], showed a fistula rate of 5.4%, a stenosis rate of 2.7%, and a wound dehiscence rate of 2.7%. Finally, the combination of VD flap with TV was employed in one group [37], with no reported postoperative complications.

**Fig. 2:**
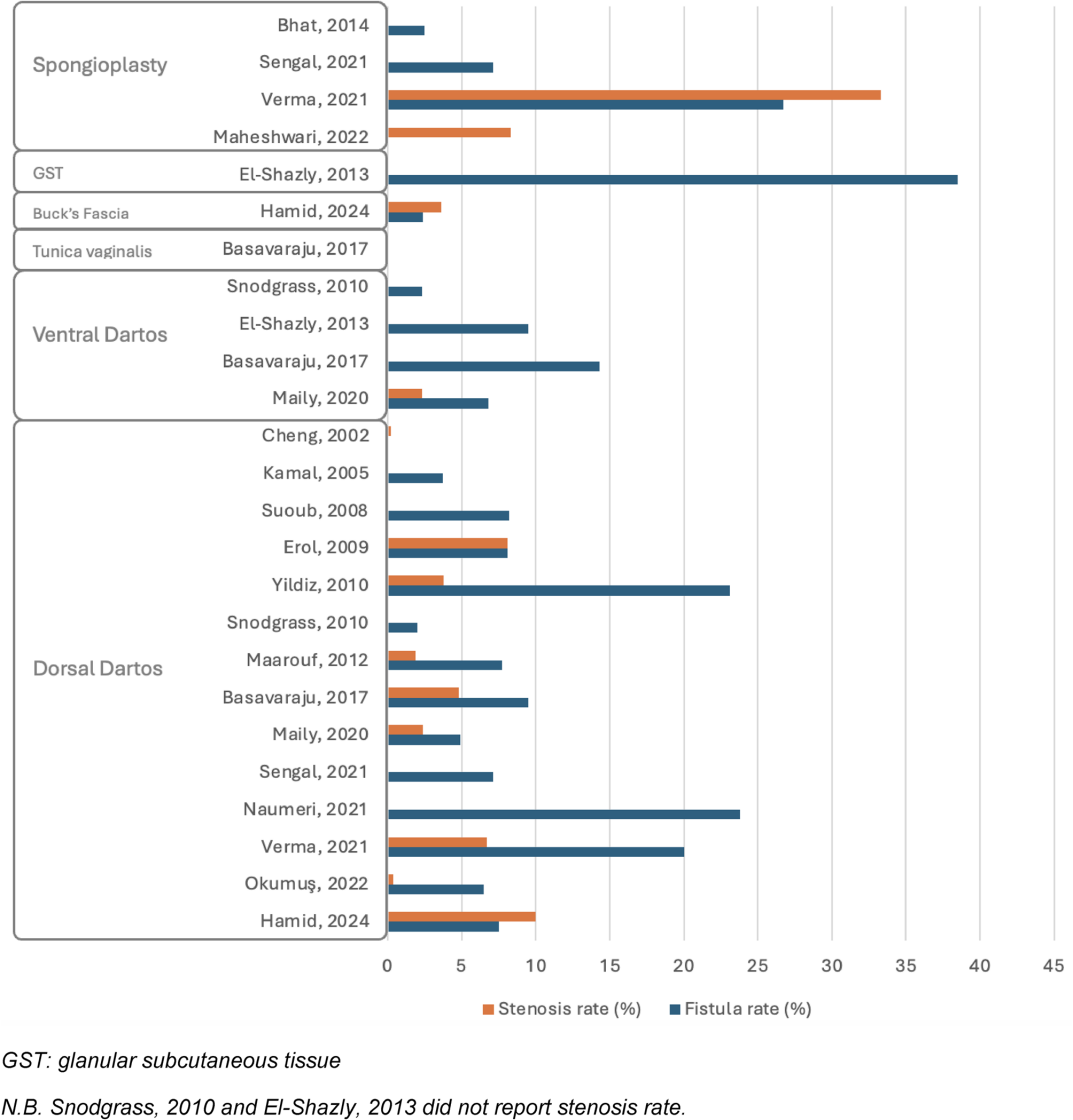
Complication rate in distal hypospadias repair using single layers. *GST* glanular subcutaneous tissue. N.B. Snodgrass, 2010 and El-Shazly, 2013 did not report stenosis rate

**Fig. 3:**
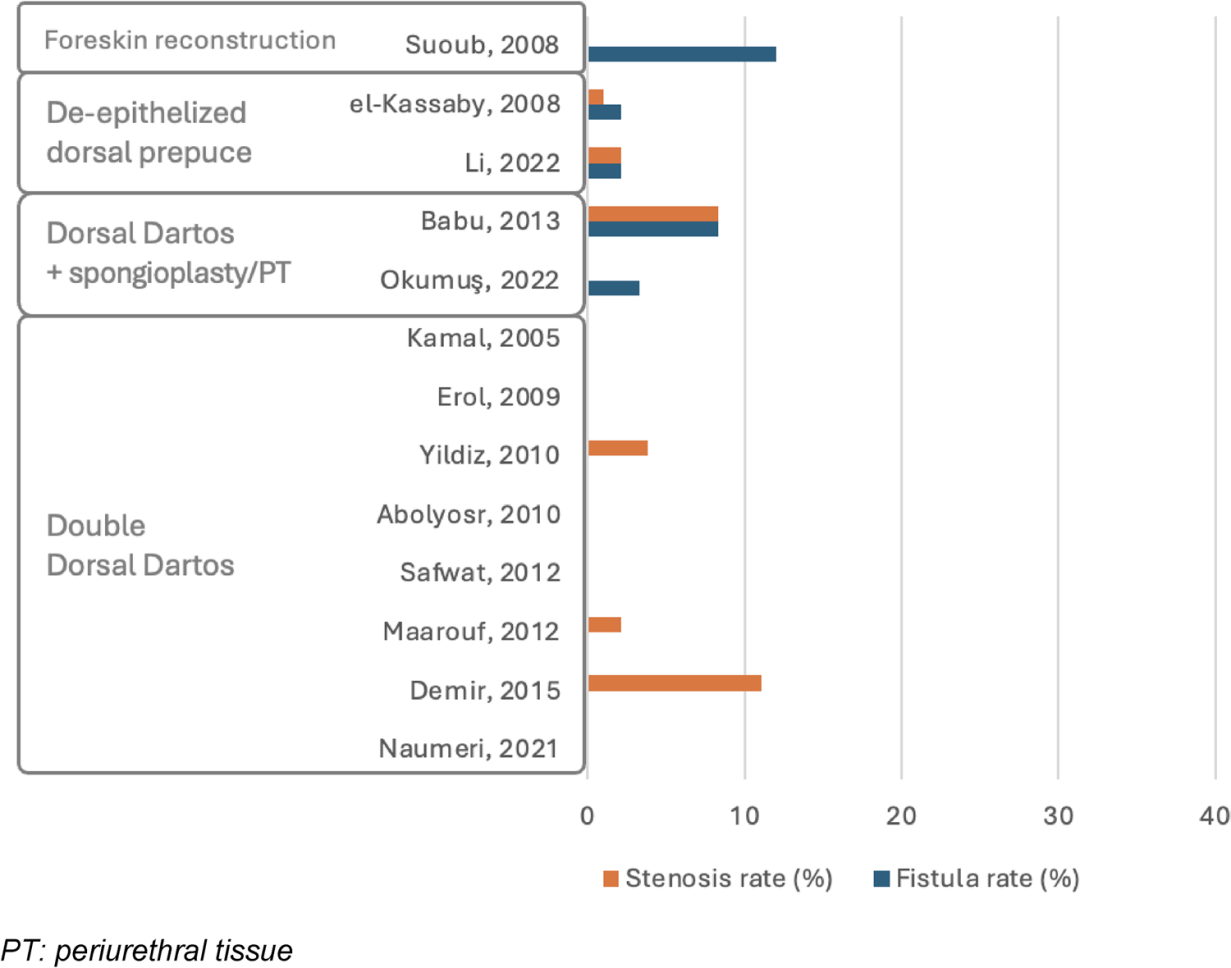
Complication rate in distal hypospadias repair using double layers. *PT* periurethral tissue

**Fig. 4:**
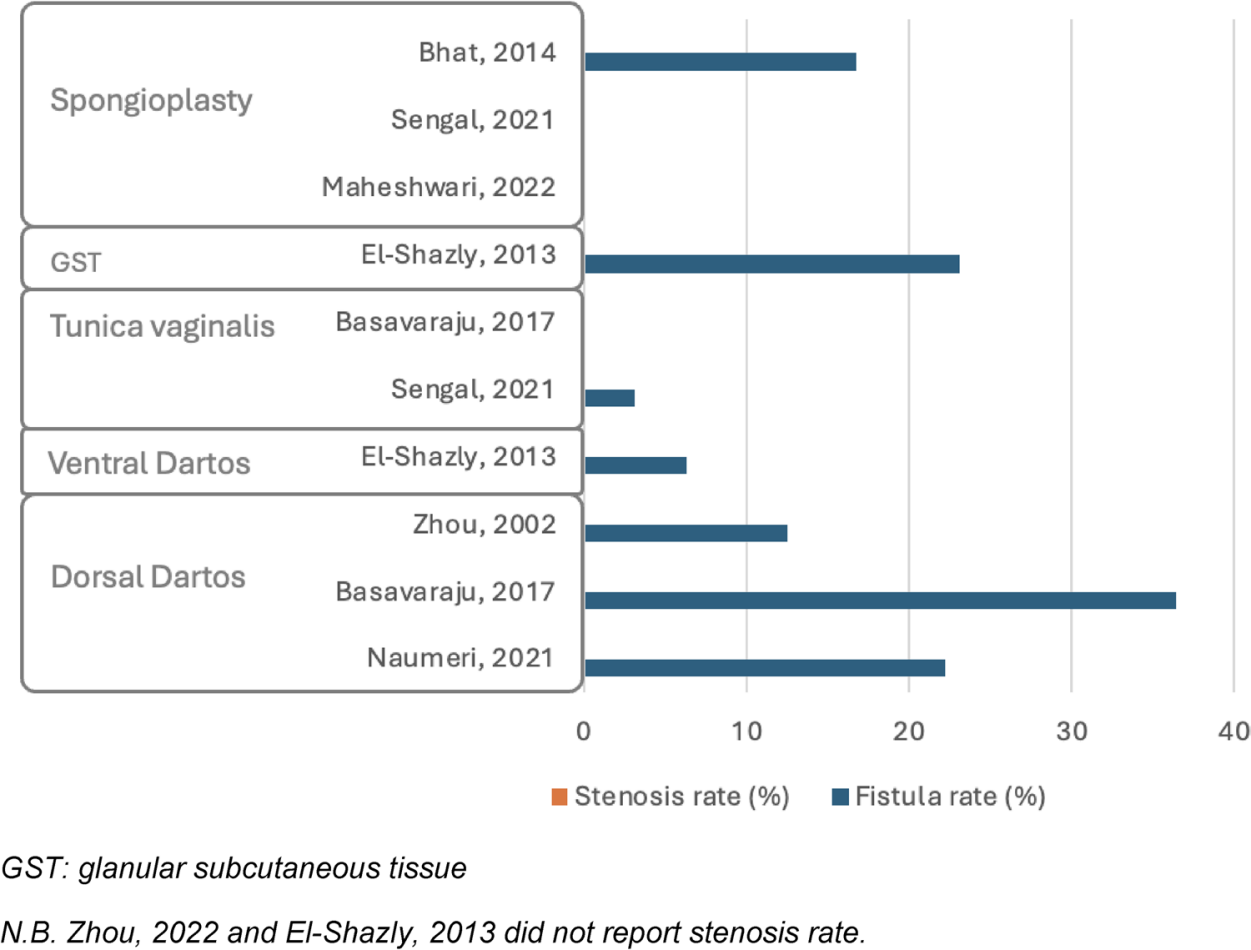
Complication rate in midpenile hypospadias repair using single layers. *GST* glanular subcutaneous tissue. N.B. Zhou, 2022 and El-Shazly, 2013 did not report stenosis rate

**Fig. 5:**
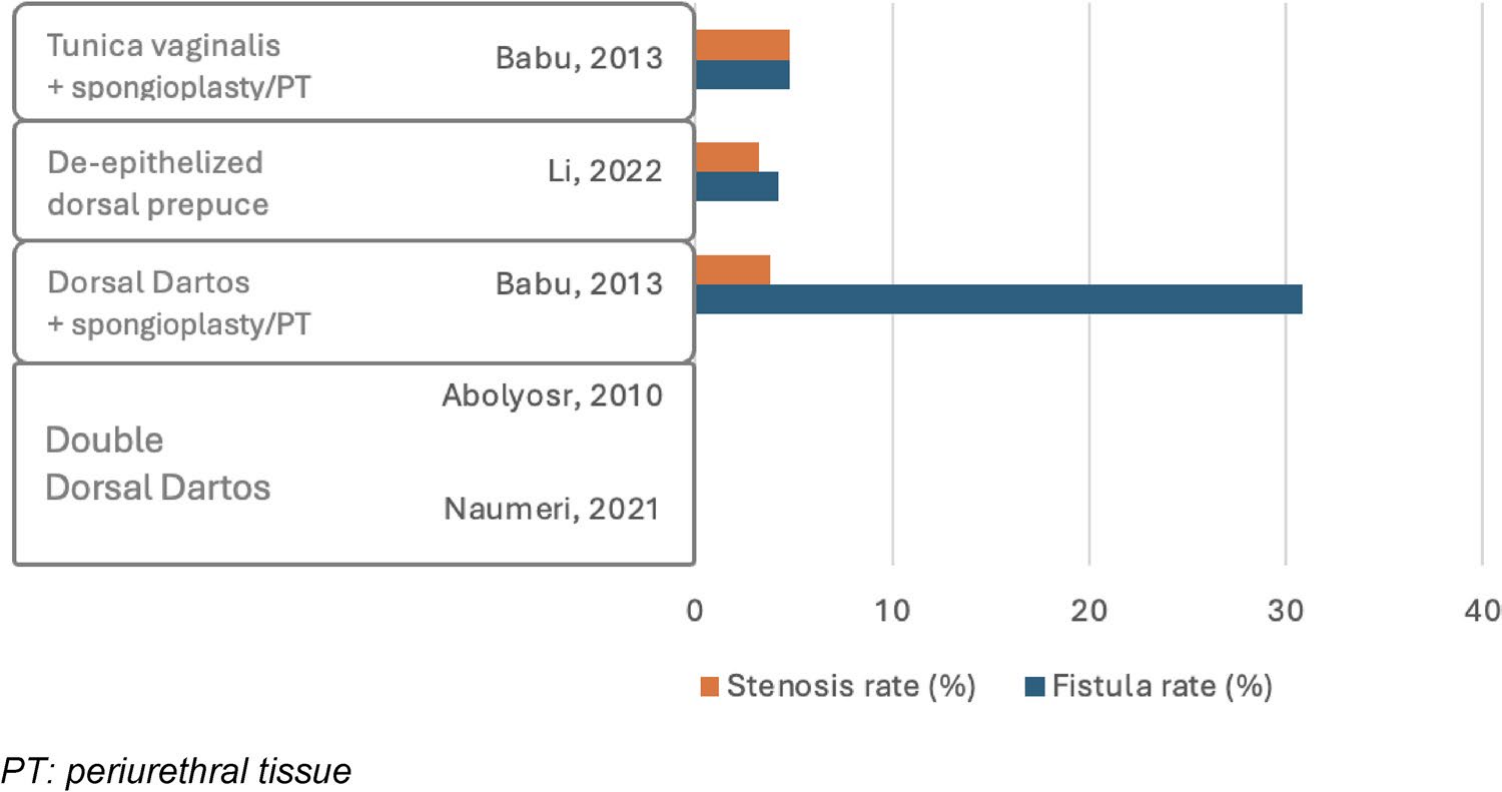
Complication rate in midpenile hypospadias repair using double layers. *PT periurethral tissue*

A summary of the main results in terms of fistula and stenosis rates, divided by hypospadias location and by the use of single or double layer, is provided in Figs. 2, 3, 4, and 5*.*

## Discussion

The use of a protective, preferably waterproof layer to shield the neourethra during hypospadias repair is widely advocated, since it enhances the success rate with low technical effort needed for tissue apposition over the neourethra [3]. By implementing this approach, the incidence of complications stays minimal, and potential UCF can be effectively addressed using readily available local tissue, alongside established adapted procedures [45].

Various forms of layers, utilized in distinct manners (single/double), are delineated in the literature. Consequently, results show significant variability between studies, and indeed, hypospadias correction encompasses numerous technical elements and variables, complicating the assessment of any singular factor as a determinant of complications.

The incidence of UCF following distal and midpenile hypospadias repair without the apposition of a second layer remains uncertain; nonetheless, it should not exceed 10% if a diligent urethroplasty is done, as suggested by Zulli et al. [46].

Despite significant limitations, several conclusions may be drawn from the literature analysis regarding the preferred second layers utilized for hypospadias repair.

The predominant type of hypospadias is distal, characterized by the meatus opening between the subcoronal area and the midshaft. This variant of hypospadias is typically corrected with a single-stage surgical procedure, except in cases where there is a significantly compromised urethral plate and/or distal urethra, sometimes associated with severe chordee [47]. In the conventional case of ‘classic’ distal hypospadias, the single DD flap is widely utilized as a second layer over the neourethra [48]. In this systematic review, 12 out of fifteen homogeneous groups treated with the single DD flap had a UCF rate of ≤ 10%, along with a reduced incidence of meatal stenosis. Wound dehiscence appeared to be rare, indicating that the thickness of the dartos does not affect the closure of the glans. Alternative second-layer approaches, such as the VD flap and Buck’ fascia, were often seldom utilized; yet studies indicate promising outcomes with these methods [13–15, 17, 19]. The VD and Buck’ fascia are accessible with minimal tissue manipulation, ideally decreasing post-operative edema and ecchymosis, but a more precise characterization of both flaps is required. Regarding the former, the anatomical limit between ventral and dorsal dartos remains ambiguous, while for the latter, the preservation of Buck’ fascia during penile degloving likely entails maintaining at least its superficial lamella, which may influence the correction of ventral chordee when present [13]. Similarly, spongiosal tissue may have certain limitations: its availability fluctuates with hypospadias, its application is less reproducible compared with dorsal darts flap, and it may prove challenging to adequately cover the entire neourethra along the glans component [5]. The review established that DD is the preferred tissue as a single second layer, demonstrating favorable viability, excellent availability, and technical reproducibility.

In our review, the double layers experience was predominantly characterized by the double DD flap, whereas other combinations of double layers, such as DD with spongioplasty and DD with periurethral tissue, were utilized less commonly.

The double DD UCF rates documented in our review were remarkable: seven out of eight groups exhibited a 0% rate, while one reported a 3.7% rate. Three out of eight groups exhibited meatal stenosis in 2–11% of cases. Meatal stenosis likely requires further consideration for the apposition of double DD, which surely occupies additional space behind the glans wing. In this regard, the meta-analysis performed by Yuan et al. [48] showed no statistically significant difference in the incidence of meatal stenosis between single and double DD flap groups, suggesting that other elements such as the thickness of the dartos, the width of the glans wings achieved during preparation, and the extent of the neourethra acquired by the second layer may influence the likelihood of this complication occurring.

Midshaft hypospadias exhibited UCF rates above those documented for the distal defect, as demonstrated in the literature [49]. In our analysis, five out of ten patient groups treated with a single layer over the neourethra for midpenile hypospadias exhibited UCF rates of 10% or less, whereas the other five had UCF rates ranging from 12.5% to 36.4%. The occurrence of fistula was notably elevated in three investigations utilizing single DD, but it was minimal (0–3.1%) when employing TV. In the meta-analysis performed by Yang et al. [50], TV flap appeared to be a better option compared to dartos flap in minimizing UCF and prepuce-related complications after TIPU. This flap is primarily utilized for proximal defect types; in this context, the TV is largely accessible, particularly when the dissection reaches the infrascrotal region. In midshaft hypospadias, TV could be a viable alternative, offering an extensive and broad secondary layer. However, potential negative impacts include additional scrotal wounds, risks of testicular and spermatic cord injury, postoperative scrotal hematoma, and testicular retraction, which should be carefully considered when planning to use this flap. The analysis did not clarify why the single DD flap, which is highly effective for distal defects, yielded unsatisfactory outcomes in midshaft instances.

The double-layer technique for midpenile hypospadias was utilized in five homogeneous groups. Only one out of five groups exhibited significantly elevated UCF rates of 30.8% with the use of DD plus spongioplasty or periurethral tissue [26]. However, in the remaining four, the incidence of UCF remained at or below 5%. It is noteworthy that whereas single DD did not yield favorable outcomes as a standalone second layer, the application of double DD in two out of five groups resulted in a low UCF rate and no meatal stenosis at follow-up. The application of double covering during urethroplasty for a midshaft lesion appears to enhance outcomes relative to the use of a single reinforcing layer, in accordance with a recent meta-analysis by Borkar et al. [4].

Sixteen patient groups across thirteen studies that did not differentiate the results between distal and midpenile forms had a single-layer coverage. Ten out of sixteen groups exhibited UCF rates of 10% or less. The dorsal and ventral dartos flaps were the most often utilized. Specifically, single DD was implemented in seven groups, with a UCF rate of ≤ 10% in five out of seven. Eight groups had a double layer over the urethroplasty within the mixed cohort. The UCF rate ranged from 0% to 5.4%, while the incidence rate for the most often utilized approach (dorsal DD) was between 0% and 2.5% in five out of eight groups. The incidence rate of meatal stenosis in the same groups with dorsal DD ranged from 2.4% to 17%.

The systematic review demonstrated significant overlap in outcomes across various patient groups. This highlights the challenge of focusing the outcomes just on neourethral coverage and the effectiveness of the applied protective layers.

The DD was the most utilized second layer, whether in single or double configuration. Despite potential variability in individual research outcomes, one should anticipate UCF rates below 10% when employing single DD or double DD for the treatment of both distal and midshaft hypospadias via single-stage TIPU repair. The application of DD may be associated with a marginally increased risk of meatal stenosis; therefore, the thickness of the dartos flap must be meticulously adjusted with the tension of the glans wings during closure.

Sometimes, hypospadias present with insufficient and poorly vascularized ventral skin. Usually, meticulous skin reconfiguration and preservation are enough to guarantee a safe closure. However, in cases of very severe ventral skin defects that may potentially lead to a gap during closure, the inner plate of the dorsal prepuce can be transferred to the ventral side to resolve the lack of skin. This maneuver allows the excision of poorly vascularized and thin skin edges and enables tension-free ventral sutures. Nevertheless, in these cases, the persistent use of double-layer DD may cause ventral wound dehiscence or skin necrosis. In such scenarios, using a single-layer DD or alternative coverage materials, such as TV, represents a potential solution to safely cover the urethroplasty.

The TV flap is efficient in achieving a low UCF rate; nevertheless, its anatomical distance in distal hypospadias likely restricts its application and, consequently, the experiences documented in the literature.

The primary limitation of this literature review is the inadequate comparability of studies with varying patient selections. This bias was alleviated, though not eradicated, by considering solely single-stage TIPU repairs. The results description was enhanced by comparing homogeneously treated patient groups rather than individual studies.

## Conclusions

The application of a protective layer over the neourethra, either singularly or doubly, is commonly recommended. Various layers may have similar outcomes. DD is the most utilized second layer due to its accessibility, offering good coverage with an overall complication rate of less than 10%, especially when a double layer is employed. Additional data are required for other tissue types, such as the VD of Buck’s fascia. The TV flap appears to yield a high success rate; nevertheless, its application is more labor-intensive for distal defects, leading to reduced adoption. A comprehensive meta-analysis of all types of layers commonly used in clinical settings, currently unreliable due to the heterogeneity of available literature and the paucity of RCTs, might be useful in the future in guiding the optimal selection of the second layer in distal and midpenile hypospadias repair.

## Data Availability

No data sets were generated or analysed during the current study.

## References

[1] van der Horst HJR, de Wall LL (2017) Hypospadias, all there is to know. Eur J Pediatr. 10.1007/s00431-017-2864-5 28190103 10.1007/s00431-017-2864-5 PMC5352742

[2] Borkar N, Tiwari C, Nair A, Das K, Sinha CK (2024) Snodgrass (tubularized incised plate) versus Mathieu repair of distal hypospadias: a systematic review and meta-analysis of randomized controlled trials. Urol J. 10.1177/03915603241273616 10.1177/03915603241273616 39171667

[3] Fahmy O, Khairul-Asri MG, Schwentner C, Schubert T, Stenzl A, Zahran MH et al (2016) Algorithm for optimal urethral coverage in hypospadias and fistula repair: a systematic review. Eur Urol. 10.1016/j.eururo.2015.12.047 26776935 10.1016/j.eururo.2015.12.047

[4] Borkar N, Tiwari C, Nair A, Sinha CK, Ratan SK, Naredi BK (2024) Single dartos flap versus double dartos flap in hypospadias repair: a systematic review and meta-analysis with trial sequential analysis and fragility index. Urologia Journal. 10.1177/03915603241231058 38345023 10.1177/03915603241231058

[5] Verma A, Murtaza S, Kundal VK, Sen A, Gali D (2021) Comparison of Dartos flap and spongioplasty in Snodgrass urethroplasty in distal penile hypospadias. World J Pediatr Surg. 10.1136/wjps-2021-000294 36474978 10.1136/wjps-2021-000294 PMC9648594

[6] Yildiz A, Bakan V (2010) Comparison of perimeatal-based flap and tubularized incised plate urethroplasty combined with single- or double-layer dartos flap in distal hypospadias. Urol Int. 10.1159/000288226 20389153 10.1159/000288226

[7] Maarouf AM, Shalaby EA, Khalil SA, Shahin AM (2012) Single vs. double dartos layers for preventing fistula in a tubularised incised-plate repair of distal hypospadias. Arab J Urol 10(4):408–413 26558059 10.1016/j.aju.2012.09.002 PMC4442928

[8] Safwat A, Al-Adl AM, El-Karamany T (2012) Vascularized dartos flap in conjunction with tubularized incised plate urethroplasty: Single versus double flaps for management of distal hypospadias. Curr Urol 6(2):67 24917716 10.1159/000343511 PMC3783336

[9] Erol A, Kayikci A, Memik O, Cam K, Akman Y (2009) Single vs. double dartos interposition flaps in preventing urethrocutaneous fistula after tubularized incised plate urethroplasty in primary distal hypospadias: a prospective randomized study. Urol Int. 10.1159/000241682 19829040 10.1159/000241682

[10] Kamal BA (2005) Double dartos flaps in tubularized incised plate hypospadias repair. Urology. 10.1016/j.urology.2005.05.020 16286134 10.1016/j.urology.2005.05.020

[11] Suoub M, Dave S, El-Hout Y, Braga LHP, Farhat WA (2008) Distal hypospadias repair with or without foreskin reconstruction: a single-surgeon experience. J Pediatr Urol. 10.1016/j.jpurol.2008.01.215 18790424 10.1016/j.jpurol.2008.01.215

[12] Cheng EY, Vemulapalli SN, Kropp BP, Pope JC IV, Furness PD, Kaplan WE et al (2002) Snodgrass hypospadias repair with vascularized dartos flap: the perfect repair for virgin cases of hypospadias? J Urol 168:1723–1726 12352344 10.1097/01.ju.0000026940.33540.31

[13] Hamid R, Baba AA (2024) Comparison of outcome of TIP urethroplasty with or without Buck’ Fascia repair. BMC Urol 24(1):133 38937743 10.1186/s12894-024-01468-x PMC11210143

[14] Snodgrass WT, Bush N, Cost N (2010) Tubularized incised plate hypospadias repair for distal hypospadias. J Pediatr Urol. 10.1016/j.jpurol.2009.09.010 19837000 10.1016/j.jpurol.2009.09.010

[15] El-Shazly M (2013) Inclusion and exclusion criteria to overcome bias and reach a valid conclusion for interpositional flap coverage in primary hypospadias repair with tubularized incised plate urethroplasty. Ann Plast Surg. 10.1097/SAP.0b013e31825516a0 22868318 10.1097/SAP.0b013e31825516a0

[16] Okumuş M, Tireli GA (2022) Tubularized incised plate repair in 473 primary distal hypospadias cases: an evaluation of outcomes according to coverages and stent types. Actas Urol Esp. 10.1016/j.acuroe.2022.01.001 35256325 10.1016/j.acuroe.2022.01.001

[17] Maily JAH, Gali S, Ghazi MJ (2020) Comparison between dorsal and ventral dartos flap interposition in a tubularized incised plate repair of distal hypospadias. Int J Pharm Res 12:3243

[18] Naumeri F, Munir MA, Ahmad HM, Sharif M, Awan NU, Butt G (2021) Comparison of urethrocutaneous fistula rate after single dartos and double dartos tubularized incised plate urethroplasty in pediatric hypospadias. Cureus. 10.7759/cureus.13378 33754103 10.7759/cureus.13378 PMC7971730

[19] Basavaraju M, Balaji DK (2017) Choosing an ideal vascular cover for Snodgrass repair. Urol Ann. 10.4103/UA.UA_90_17 29118537 10.4103/UA.UA_90_17 PMC5656960

[20] Sengol J, Gite VA, Agrawal M, Sankapal P, Shaw V (2021) Choosing an ideal second layer cover in snodgrass repair for various types of hypospadias. Turk J Urol 47(3):229–236 35929877 10.5152/tud.2021.20421 PMC8260083

[21] Zhou Y, Lu J, Takahashi G (2002) Snodgrass procedure for primary hypospadias repair. Int J Urol. 10.1046/j.1442-2042.2002.00455.x 12010316 10.1046/j.1442-2042.2002.00455.x

[22] Bhat A, Sabharwal K, Bhat M, Saran R, Singla M, Kumar V (2014) Outcome of tubularized incised plate urethroplasty with spongioplasty alone as additional tissue cover: a prospective study. Indian J Urol. 10.4103/0970-1591.134234 25378820 10.4103/0970-1591.134234 PMC4220378

[23] Maheshwari M, Gite VA, Agrawal M, Sankapal P, Shaw V, Sharma S et al (2022) Outcome of spongioplasty alone as second layer of tubularised incised plate urethroplasty in patients with hypospadias. Afr J Urol. 10.1186/s12301-022-00305-7

[24] Demir A, Karadaʇ MA, Çeçen K, Uslu M, Arslan ÖE (2015) Our experience with a double-layer surgical technique for preventing fistula development in children and young adults with hypospadias. Urol Int 95(2):132–136 26044984 10.1159/000431103

[25] El-Kassaby AW, Al-Kandari AM, Elzayat T, Shokeir AA (2008) Modified tubularized incised plate urethroplasty for hypospadias repair: a long-term results of 764 patients. Urology. 10.1016/j.urology.2007.11.121 18295308 10.1016/j.urology.2007.11.121

[26] Babu R, Hariharasudhan S (2013) Tunica vaginalis flap is superior to inner preputial dartos flap as a waterproofing layer for primary TIP repair in midshaft hypospadias. J Pediatr Urol. 10.1016/j.jpurol.2012.10.022 23186594 10.1016/j.jpurol.2012.10.022

[27] Abolyosr A (2010) Snodgrass hypospadias repair with onlay overlapping double-layered dorsal dartos flap without urethrocutaneous fistula: experience of 156 cases. J Pediatr Urol. 10.1016/j.jpurol.2009.09.012 19857999 10.1016/j.jpurol.2009.09.012

[28] Li J, Li S, Yang Z, Ke Z, Zhang T, Yin J (2022) A simple technique to repair distal and mid-shaft hypospadias using a de-epithelialized Byars’ flap. J Int Med Res. 10.1177/03000605221115150 35999815 10.1177/03000605221115150 PMC9421228

[29] Al-Taher R, Nofal M, Yousef AJ, Rashdan M, Tarawneh A, Alsmadi J et al (2023) Double dartos flap layer in tubularized incised plate urethroplasty to prevent urethrocutaneous fistula in uncircumcised patients with distal hypospadias. Asian J Androl 25(1):93-97/35975363 10.4103/aja202251 PMC9933958

[30] Tabassi KT, Mohammadi S (2010) Tunica vaginalis flap as a second layer for tubularized incised plate urethroplasty. Urol J 7(4):254–257 21170855

[31] Bilici S, Sekmenli T, Gunes M, Gecit I, Bakan V, Isik D (2011) Comparison of dartos flap and dartos flap plus spongioplasty to prevent the formation of fistulae in the snodgrass technique. Int Urol Nephrol. 10.1007/s11255-011-9943-8 21442394 10.1007/s11255-011-9943-8

[32] Jia W, Liu G, Zhang L, Wen Y, Fu W, Hu J et al (2016) Comparison of tubularized incised plate urethroplasty combined with a meatus-based ventral dartos flap or dorsal dartos flap in hypospadias. Pediatr Surg Int. 10.1007/s00383-016-3860-y 26783086 10.1007/s00383-016-3860-y

[33] Mahmoud AY, Gouda S, Gamaan I, Baky Fahmy MA (2019) Autologous platelet-rich plasma covering urethroplasty versus dartos flap in distal hypospadias repair: a prospective randomized study. Int J Urol. 10.1111/iju.13912 30719774 10.1111/iju.13912

[34] Omar RG, Khalil MM, Sherif H, Elezaby H (2018) Pedicled preputial island flap for double functions in hypospadias surgery. Turk J Urol 44(5):423–427 29799409 10.5152/tud.2018.49035 PMC6134987

[35] Soygur T, Arikan N, Zumrutbas AE, Gulpinar O (2005) Snodgrass hypospadias repair with ventral based dartos flap in combination with mucosal collars. Eur Urol. 10.1016/j.eururo.2005.02.022 15925087 10.1016/j.eururo.2005.02.022

[36] Dahal S, Mahat B, Hussain N, Saleem M, Rahman U (2022) Buck’s fascia in addition to Dartos fascia is an effective intermediate layer in repair of hypospadias. Pak J Med Health Sci. 10.53350/pjmhs20221611307

[37] Han JH, Song SH, Lee JS, Park S, Kim SJ, Kim KS (2020) Efficacy of additional tunica vaginalis flap coverage for protecting against urethrocutaneous fistulas in tubularized incised plate urethroplasty: a prospective, randomized controlled trial. Investig Clin Urol. 10.4111/icu.20200024 32734722 10.4111/icu.20200024 PMC7458875

[38] Yiğit D, Avlan D (2022) Dorsal versus ventral dartos flap to prevent fistula formation in tubularized incised plate urethroplasty for hypospadias. Urol J 19(4):315–319 35762080 10.22037/uj.v19i.7098

[39] Asad S, Khan FA, Ali S, Khan H, Rafaqat H, Khattak IUD (2023) Snodgrass hypospadias repair at ayub teaching hospital: an audit of complications and outcomes. J Ayub Med Col 35(2):259–264 10.55519/JAMC-02-11003 37422817

[40] Thomas DT, Karadeniz Cerit K, Yener S, Kandirici A, Dagli TE, Tugtepe H (2015) The effect of dorsal dartos flaps on complication rates in hypospadias repair: a randomised prospective study. J Pediatr Urol. 10.1016/j.jpurol.2014.07.010 25218352 10.1016/j.jpurol.2014.07.010

[41] Appignani A, Prestipino M, Bertozzi M, Nardi N, Falcone F (2009) Double-cross flap protection: new technique for coverage of neourethra in hypospadias repair. J Urol. 10.1016/j.juro.2009.06.054 19683748 10.1016/j.juro.2009.06.054

[42] Bertozzi M, YIldIz A, Kamal B, Mustafa M, Prestipino M, Yiǧiter M et al (2011) Multicentric experience on double dartos flap protection in tubularized incised plate urethroplasty for distal and midpenile hypospadias. Pediatr Surg Int 27(12):1331–1336 21935592 10.1007/s00383-011-2978-1

[43] Bertozzi M, Nardi N, Prestipino M, Melissa B, Magrini E, Appignani A (2016) Is the double cross flap technique the panacea for avoiding fistula formation in hypospadias surgery? Ann Pediatr Surg. 10.1097/01.XPS.0000489164.33691.5f

[44] Essam T, Hassan I, Elwagdy S (2011) Does double-layer vascularized dartos flap with snodgrass hypospadias repair improve results? UroToday Int J 4(6):69

[45] Choudhury P, Saroya KK, Jain V, Yadav DK, Dhua AK, Anand S et al (2023) ‘Waterproofing layers’ for urethrocutaneous fistula repair after hypospadias surgery: evidence synthesis with systematic review and meta-analysis. Pediatr Surg Int. 10.1007/s00383-023-05405-1 37010625 10.1007/s00383-023-05405-1

[46] Zulli A, Mantovani A, Gigola F, Landi L, Taverna M, Cini C et al (2025) Incidence of urethrocutaneous fistula after distal and midshaft hypospadias repair does not differ among patients treated with or without a protective second-layer: single tertiary centre experience. Pediatric Surg Int 41:29 10.1007/s00383-024-05926-3 39680159

[47] Bocchino AC, Cocci A, Pezzoli M, Elia A, Landi L, Mantovani A et al (2024) Long-term functional, sexual, and cosmetic outcomes in adult patients who underwent hypospadias repair during childhood at a highly specialized Italian Pediatric Hospital. Urol Res Pract 50(2):127–133 39128083 10.5152/tud.2024.24005 PMC11232063

[48] Yuan Y, Wang YW, Liang YN, Wang YY, Ho JJ, Peng TY et al (2023) A meta-analysis: single or double dartos flap layer in tubularized incised plate urethroplasty to prevent urethrocutaneous fistula? Front Pediatr. 10.3389/fped.2023.1091242 37360362 10.3389/fped.2023.1091242 PMC10286861

[49] Pfistermuller KLM, McArdle AJ, Cuckow PM (2015) Meta-analysis of complication rates of the tubularized incised plate (TIP) repair. J Pediatr Urol. 10.1016/j.jpurol.2014.12.006 25819601 10.1016/j.jpurol.2014.12.006

[50] Yang H, Xuan X, Hu D, Zhang H, Shu Q, Guo X et al (2020) Comparison of effect between dartos fascia and tunica vaginalis fascia in TIP urethroplasty: a meta-analysis of comparative studies. BMC Urol. 10.1186/s12894-020-00737-9 33059661 10.1186/s12894-020-00737-9 PMC7559339

